# Annotations

**Published:** 1903-04-25

**Authors:** 


### Annotations.

### The St. Francis Leper Guild.

The methods of the League of Mercy seem to have been taken to heart by the organisers of the "St. Francis Leper Guild." Members of the Guild are asked to contribute themselves a shilling a year, and to collect subscriptions of a penny a year from every available source. The shillings of the members of the Guild are to be used for working expenses, but the "Leper Penny" is to be kept intact for the help of the lepers. The organisation dates from the year 1897, and as is very natural, when one remembers how Father Damien gave his life for lepers, is largely supported by the Catholic Church. The Pope and the Archbishops of Westminster and Paris have given their approval of the work. But as leprosy owns no creed, there is no reason why Protestants should refrain from helping in it. There are at least half a million lepers in India alone, which may well give the work of the Guild a claim on the attention of Britain. The Duke of Westminster recently lent Grosvenor House for a concert in its aid, and a "Spring Flower Ball" is to be held for the same object in the Empress Rooms on May 8th. The object of the Guild is primarily the segregation of lepers into colonies where not only can they be watched and tended, but where the disease can be studied, and where, it is hoped, it may be shown that when proper precautions are taken leprosy is no more contagious than other diseases. It is intended also to give employment to adult lepers, until their disease reaches an advanced stage, which will help to keep them from the depression incident to their malady, and will also be useful to the colony, while the healthy children of lepers will be taught in schools where they will be closely watched, and separated from their companions if the disease manifests itself. The Guild hopes also to give special training to sisters of charity who are willing to devote their lives to the care of the lepers. Grants of money are given to existing leper colonies, the largest sum voted last year for any place—£40—having been sent to one at Gotemba, in Japan. If the fever of enthusiasm and sympathy which was aroused by the tale of Father Damien's life and death has not wholly burned itself out, the St. Francis Leper Guild should not fail of success.

### Politics, Patronage, and the Poor.

Those inhabitants of the State of New York who take an unselfish interest in the poor and suffering are much grieved by a law which has lately been passed there. By this the entire management of all State charities, including asylums for the insane, almshouses, industrial schools, and the like, is put under the direct control of the State. Hitherto these institutions have been largely managed by local committees, consisting of voluntary workers, who were moved by a real interest in these unfortunate wards of the State. By a new Act of the legislature all this is changed, and all the State charities will now be under direct political domination. As Mr. Eugene Philbin said at a State conference on the subject of "Charities and Correction" among experienced workers in practical charity, no word is more pregnant with offensive meanings than "politics." In America the effect of political influence on philanthropic institutions is much more serious than it is here, though, even in this country, there occur cases where, in an appointment, not the best man, but the man with most influence is sure to be successful. But, at any rate, the appointment is *aut vitam aut culpam*, and an honest man, even if he knew little of the work to which he was set to begin with, has a chance of learning it before his life is past. But in America every new Cabinet appoints its own supporters to the smallest posts as well as to the greatest, and there is a temptation for the place-holder, knowing that he has only a few years of power before him, to make all he can out of it, and troubles very little about the duties he is supposed to perform. Indeed, there is always a danger that the political nominee may ignore the reason of his existence. Naturally there are many who are above selfish considerations, but on the whole the voluntary committee of men and women who have no "axe to grind" is the best corrective. Although our charities in this country are not in such danger of being ruined by political influence as those of America, we are by no means free from similar risks, and it is well to remind those who would see everything under State control that in the places where this is most general it is most disliked by genuine philanthropists.

### Notification in Excelsis.

It has been reserved for the Island of Malta to go "one better" than most places in the way of notification. In this island, when any patient is feverish for more than ten days, it is the duty of his medical attendant to report the case to the public health authorities. The latter then examine into matters for themselves, and if no sign of tubercle, enteric, or other condition that is commonly accompanied by pyrexia can be discovered, the case is put down as one of "Mediterranean" or "undulant" fever. Elsewhere this fever is often more briefly described as "Malta fever," but in Malta itself this is not considered at all polite. Certainly the disease has now been identified in China, Upper India, and in many parts of the Mediterranean besides Malta, thanks to the ease with which cultures of the M. meletensis can be carried about, and the simplicity in this fever of the application of the Widal process. In no community, however, does it play so prominent a part as it does in the island in which it was first distinguished from malaria and enteric. It is a remarkable disease, and probably no other produces such acute illness with so few appearances during the latter intermittent periods of constitutional disturbance. The cachexia, however, which in many cases it eventually produces, is often very extreme, and no disease is less amenable to treatment. In matters hygienic, other than notification, Malta can hardly be called progressive, general sanitation being decidedly behind the times, and its quarantine regulations being so severe as to have been a very weak point in England's "case" at sundry international conferences. For Foreign Delegates have been able to meet England's arguments in favour of abbreviating quarantine and decreasing its severity by reference to the line adopted in islands under her own nominal administration.

